# The impact of immune dysregulation on the risk of malignancy in common variable immunodeficiency: insights from a multicenter study

**DOI:** 10.3389/fimmu.2024.1465159

**Published:** 2024-10-16

**Authors:** Marta Dafne Cabañero-Navalon, Victor Garcia-Bustos, Héctor Balastegui-Martin, Carmen Bracke, Lourdes Mateu, Xavier Solanich, Juan Luis Carrillo-Linares, Angel Robles-Marhuenda, Francesc Puchades, Ana Pelaez Ballesta, Nuria Lopez-Osle, Miguel Ángel Torralba-Cabeza, Ana María Bielsa Masdeu, Jorge Gil Niño, Nuria Tornador Gaya, Guillem Pascual Castellanos, Rosario Sánchez-Martínez, José Manuel Barragán-Casas, Andrés González-García, José Luis Patier de la Peña, Daniel López-Wolf, Antonia Mora Rufete, Alba Canovas Mora, Pedro Moral Moral

**Affiliations:** ^1^ Primary Immunodeficiencies Unit, Department of Internal Medicine, University and Polytechnic Hospital La Fe, Valencia, Spain; ^2^ Research Group of Chronic Diseases and HIV Infection, Health Research Institute La Fe, Valencia, Spain; ^3^ Severe Infection Research Group, Health Research Institute La Fe, Valencia, Spain; ^4^ Infectious Diseases Service, Germans Trias i Pujol Hospital, Badalona, Spain; ^5^ Fight Infections Foundation, Germans Trias i Pujol Hospital, Badalona, Spain; ^6^ Internal Medicine Department, Hospital Universitari de Bellvitge, L’Hospitalet de Llobregat, Barcelona, Spain; ^7^ Translational Medicine Area, Institut d’Investigació Biomèdica de Bellvitge (IDIBELL), Barcelona, Spain; ^8^ Department of Clinical Sciences, Faculty of Medicine and Health Sciences, Universitat de Barcelona, Barcelona, Spain; ^9^ Department of Internal Medicine, Virgen de la Victoria University Hospital, Málaga, Spain; ^10^ Department of Internal Medicine, La Paz University Hospital, Madrid, Spain; ^11^ Department of Internal Medicine, University General Hospital of Valencia, Valencia, Spain; ^12^ Department of Internal Medicine, Rafael Méndez University Hospital, Murcia, Spain; ^13^ Department of Internal Medicine, Cruces University Hospital, Bizkaia, Bilbao, Spain; ^14^ Unit for Rare Diseases, Internal Medicine Service, Lozano Blesa University Hospital, Zaragoza, Spain; ^15^ Department of Internal Medicine, Miguel Servet University Hospital, Zaragoza, Spain; ^16^ Immunodeficiencies clinic, Internal Medicine Department, 12 de Octubre Hospital, Madrid, Spain; ^17^ Department of Internal Medicine, University General Hospital of Castellón, Castellón, Castellon, Spain; ^18^ Internal Medicine Department, Dr. Balmis General University Hospital, Instituto de Investigación Sanitaria y Biomédica de Alicante (ISABIAL), Alicante, Spain; ^19^ Department of Internal Medicine, Complejo Asistencial de Ávila, Ávila, Spain; ^20^ Systemic Autoimmune Diseases Unit, Internal Medicine Service, Ramón y Cajal Hospital, Instituto Ramón y Cajal de Investigación Sanitaria (IRYCIS), Madrid, Spain; ^21^ Department of Internal Medicine, University Hospital Alcorcón Foundation, Madrid, Spain; ^22^ Department of Internal Medicine, General University Hospital of Elche, Alicante, Spain

**Keywords:** common variable immunodeficiency, immune dysregulation, malignancy, cancer risk, immunosuppressants

## Abstract

**Background:**

Common Variable Immunodeficiency (CVID) represents a heterogenic group of primary immunodeficiencies (PID) characterized by impaired antibody production and susceptibility to infections. Non-infectious complications, such as autoimmune diseases, lymphoproliferative disorders, and malignancies, now significantly impact prognosis. Moreover, both hematologic and solid organ malignancies are more frequently observed in CVID patients compared to other PIDs. The risk factors for carcinogenesis in CVID remain largely unknown.

**Objective:**

This multicenter study aims to characterize the clinical profile of cancer in CVID patients in Spain and to identify independent risk factors associated with malignancy development, focusing on the role of immune dysregulation.

**Methods:**

A nationwide, cross-sectional study was conducted from November 2019 to May 2022, involving 17 hospitals treating PID patients in Spain. Data were collected systematically on demographics, infectious and non-infectious comorbidities, immunological parameters, and treatment. Statistical analysis, including multivariate logistic regression, was performed to identify risk factors associated to malignancy.

**Results:**

Of 250 CVID patients, 38 (15.26%) were diagnosed with cancer, predominantly non-Hodgkin lymphoma, gastric cancer, and lung adenocarcinoma. Cancer patients were significantly older (mean age 60.70 vs. 49.36 years, p<0.001) and had higher rates of immune dysregulation (81.58% vs. 59.7%, p=0.01). Immune dysregulation was an independent risk factor for cancer (OR 2.19, p=0.04), alongside previous immunosuppressant therapy (OR 2, p=0.031), higher IgM levels (OR 1.008 per SD, p=0.012), older age (OR 1.04, p<0.001), and lower CD4 cell counts at diagnosis (OR 0.997, p<0.001).

**Conclusions:**

This study highlights the increased cancer risk in CVID patients, with immune dysregulation, prior immunosuppressant use, elevated IgM levels, and lower CD4 cell counts as conjointly associated. These findings underscore the need for vigilant cancer screening and tailored management strategies in CVID patients to improve outcomes. Future research should focus on elucidating the molecular mechanisms linking immune dysregulation and malignancy in CVID.

## 1 Introduction

Common variable immunodeficiency (CVID) constitutes a heterogeneous group of primary immunodeficiency disorders (PID) with an estimated prevalence of 1:50,000 to 1:25,000 (1, 2). It is characterized by decreased levels of serum IgG, along with decreased IgM and/or IgA, after excluding secondary causes of hypogammaglobulinemia (1, 2). Historically, infectious diseases were the primary cause of morbidity and mortality in CVID patients until the introduction of immunoglobulin replacement therapy (IgRT) in the late 20th century. This treatment has significantly reduced infection-related complications, shifting the burden towards non-infectious complications such as autoimmune diseases, benign lymphoproliferative disorders, and cancer, which now have a larger impact on prognosis (3, 4). These immune dysregulation-related phenomena may affect up to 70% of patients, contributing to an 11-fold increased risk of death (5). Notably, IgRT does not seem to prevent or improve many of these conditions (5).

Moreover, both hematologic and solid organ malignancies are more frequently observed in CVID patients compared to other PIDs, and they are associated with poorer outcomes (3, 6). Various studies report a variable frequency of malignancy in CVID patients (3, 5–9), with the incidence of cancer around 10% (ranging from 1.5% to 20.7%). These malignancies typically occur during the 4th to 6th decades of life, with a risk 5-12 times higher than the general population (5, 10). The most frequently reported malignancies in CVID patients include non-Hodgkin lymphoma (NHL), gastric carcinoma, and leukemia (3, 5–7, 9).

The risk factors for carcinogenesis in CVID remain largely unknown. It is hypothesized that immune dysregulation may be associated with the development of neoplasia in this population (11–13). Several specific manifestations of this dysimmunity have been identified as potential risk factors for malignancy, such as the history of immune thrombocytopenic purpura with over a threefold increase in cancer risk (14), or the presence of arthritis, atrophic gastritis, or interstitial lung disease (ILD) (17).

Currently, there are no standardized protocols for cancer screening in CVID patients, nor are there validated tools to accurately assess the risk of neoplasia development in this population (17). The heterogeneity of CVID and its associated complications complicate the establishment of universal screening guidelines (7). Furthermore, the interplay between immune dysregulation and cancer development remains poorly understood, necessitating comprehensive research to elucidate these mechanisms.

The aim of this study was to characterize the clinical profile of cancer patients in CVID in Spain, and to identify the potential independent risk factors associated to its presence, exploring the impact of the immune dysregulation subpopulation and its potential role in malignancy.

## 2 Materials and methods

### 2.1 Study design, setting and population

A multicenter, cross-sectional, nationwide study of patients diagnosed with CVID was conducted in Spain from November 2019 to May 2022. Seventeen hospitals treating PIDs participated in establishing the GTEM-SEMI-CVID Registry, an initiative led by the Working Group of Rare Diseases of the Spanish Society of Internal Medicine (GTEM-SEMI). Patients aged 16 years and older with a confirmed diagnosis of CVID, according to the ESID working definitions (2), who were currently or had previously been under follow-up by the participating units, were eligible for inclusion. Patients with confirmed monogenic immunodeficiencies were excluded from eligibility.

### 2.2 Data collection and variables

The GTEM-SEMI-CVID-Registry systematically compiles comprehensive data on patients, including sociodemographics, epidemiology, genetics, comorbidities, imaging, laboratory results, treatments, and outcomes, as in Cabañero-Navalon et al. (7). Demographic information included sex, age at diagnosis, clinical onset, diagnostic delay, and follow-up duration. Genomic data, family history, and consanguinity were recorded. Infectious complications were registered, including major bacterial, opportunistic, and chronic infections. Non-infectious comorbidities included autoimmune cytopenias, organomegalies, systemic autoimmune disorders, and malignancies. Laboratory parameters such as immunoglobulin levels, lymphocyte subpopulations cell counts, and autoantibody presence were gathered, as well as the histopathological analyses of biopsied tissues. Therapeutic variables as IgRT and immunosuppressant use were noted. Further information on the GTEM-SEMI-CVID-Registry methodology can be found elsewhere (7).

### 2.3 Definitions

In this study examining variables associated to malignancy, cases were defined as patients who, at any point during their clinical follow-up, had a history of either hematologic and solid tumors, excluding basal cell and squamous cell skin carcinomas. Controls were patients without these conditions who mainly suffered infectious complications (iCVID).

Patients were screened for immune dysregulation (dCVID), defined by the presence of lymphadenopathy, immune cytopenias, non-infectious interstitial lung disease, splenomegaly, hepatomegaly, hepatic nodules, autoimmune organ-specific or systemic diseases, non-infectious enteropathy, and/or immune-mediated skin involvement.

### 2.4 Statistical analysis

The statistical analysis was performed using R software, version 4.3.0. The following information was considered as potentially associated to malignancy occurrence: sex, age, immunosuppression, immune dysregulation, IgG at diagnosis and last follow-up, IgM levels at diagnosis and last follow-up, IgA levels at diagnosis and last follow-up, CD4 cell count at diagnosis and last follow-up, CD8 cell count at diagnosis and last follow-up, and CD19 cell count at diagnosis and last follow-up. Median-based imputation was used to estimate missing values. Chi-square test was used to test whether the frequency of malignancy occurrence differed when patients were grouped based on gender, immunosuppression, and immune dysregulation. In addition, we aimed to explore the effect of immune dysregulation in this assessment. Therefore, for each continuous variable, a two-way ANOVA with iCVID and dCVID and malignancy was conducted. Effects or interactions with p-value below 0.05 were considered statistically significant. Partial-eta squared was used to measure the size of the effect of each main effect and interaction. Tukey *post-hoc* test (Bonferroni corrected when necessary) was applied when significant effects and interactions were found.

We used a multivariate logistic regression to study the likelihood of malignancy occurrence based on the different variables that showed significant Malignancy effect. Categorical variables showing significant relationship with malignancy were also included in the logistic regression. From the coefficients of the model, we calculated the Odds-ratio (OR), their confidence interval and the p-value associated to each coefficient (Wald test). We also studied the model’s performance by extracting the accuracy, sensibility, specificity, and the area under the receiver operating characteristic (ROC) curve (AUC). These performance parameters were extracted by using a leave-one-out cross-validation method. A nomogram was then created to represent the estimated probability of cancer at a given time for illustrative purposes.

### 2.5 Ethical statement

The development and protocol of the GTEM-SEMI-CVID Registry received independent approval from the Ethical Committees of all participating hospitals, each under their respective registry codes. The study was conducted in accordance with the Declaration of Helsinki and adhered to the STROBE guidelines. Anonymity and data confidentiality for all included patients were maintained in compliance with Spanish regulations governing observational studies.

## 3 Results

### 3.1 Study population

Out of a total of 250 patients included in the GTEM-SEMI-CVID Registry, 249 had been assessed for cancer prevalence. Among these, 38 (15.26%) were diagnosed with cancer during follow-up and were referred to as cases. Non-Hodgkin B lymphoma was the most frequent malignancy, occurring in 11 patients (4.41%). Gastric cancer and lung adenocarcinoma were reported in 5 (2.01%) and 3 (1.20%) patients, respectively. There were 2 cases of colorectal cancer, and 1 case each of breast and prostate cancer. Additionally, 3 patients had basal cell carcinoma, with 1 patient having both basal cell and squamous cell carcinoma of the skin. The remaining 7 cases included myeloma, splenic lymphoma, and cancers of the thyroid, kidney, uterus, and cervix.

All 13 patients with lymphoid malignancies showed immune dysregulation, with it being the first clinical symptom in 3 cases and malignancy in 2 at CVID debut. All five patients with gastric cancer also had immune dysregulation, and autoimmunity was the first symptom in two of these cases. Helicobacter pylori was present in 3, absent in 1, and never tested in the last. Atrophic gastritis was also present in those H. pylori positive patients.

Cases were significantly older than controls, with a mean age of 60.70 (SD 17.56) vs. 49.36 (17.88) years (p<0.001). There were no differences in sex distribution between the groups. No significant differences were seen in the diagnostic delay of CVID among groups. Immune dysregulation was present in 81.58% of cases compared to 59.7% of controls (p=0.01). Specifically, 50% of cancer patients had a history of cytopenia, compared to 30% of controls (p=0.02). Lymphadenopathy was more common in cases (57.9% vs. 30.8%, p=0.003), as was immune-mediated skin involvement (47.4% vs. 23.2%, p=0.003). There were no differences in the prevalence of splenomegaly, hepatomegaly, liver nodules, non-infectious interstitial lung disease, autoimmune diseases, or non-infectious enteropathy between the groups. Further details can be found in **Table 1**.

**Table 1:** Main characteristics of CVID patients with and without malignancy in the Spanish GTEM-SEMI-CVID cohort.

Variable		Malignancy Mean (SD) – n (%)	No malignancy Mean (SD) – n (%)	P-value
Age		60.63 (17.56)	49.36 (17.88)	4x10^-4^*
Sex	Male	16 (42.11)	105 (49.76)	0.481
	Female	22 (57.89)	106 (50.24)	
Immune dysregulation		31 (81.58)	126 (59.72=	0.01*
	Cytopenias	19 (50)	64 (30.33)	0.025*
	Lymphadenopathies	22 (57.89)	65 (30.81)	0.003*
	Splenomegaly	16 (42.11)	65 (30.81)	0.193
	Hepatomegaly	7 (18.42)	39 (18.48)	0.185
	Liver nodules	4 (10.53)	12 (5.69)	0.219
	Lung disease	26 (68-42)	121 (57.35)	0.282
	Enteropathy	14 (36.84)	69 (32.70)	0.71
	Atrophic gastritis	8 (21.05)	24 (11.37)	0.203
	Autoimmune systemic disease	8 (21.05)	41 (19.43)	0.853
	Skin affectation	17 (44.74)	49 (23.22)	0.002*
Immunoglobulins				
	IgG levels at diagnosis (mg/dL)	391.56 (238.539	405.36 (215.47)	0.475
	Last follow-up IgG levels (mg/dL)	850.07 (313.77)	852.35 (235.26)	0.982
	IgM levels at diagnosis (mg/dL)	52.32 (79.34)	53.48 (90.78)	0.720
	Last follow-up IgM levels (mg/dL)	72.31 (89.00)	67.58 (224.77)	0.164
	IgA levels at diagnosis (mg/dL)	39.09 (58.42)	48.56 (81.43)	0.976
	Last follow-up IgA levels (mg/dL)	122.29 (516.84)	66.57 (166.60)	0.810
Lymphocyte cell count				
	CD4 cell count at diagnosis (cell/mm^3^)	468.00 (243.41)	715.15 (442.60)	0.002*
	Last follow-up CD4 cell count (cell/mm^3^)	596.83 (302.48)	1,054.21 (4,228.46)	0.596
	CD8 cell count at diagnosis (cell/mm^3^)	419.45 (216.18)	537.04 (412.97)	0.604
	Last follow-up CD8 cell count (cell/mm^3^)	478.33 (243.00)	571.90 (429.06)	0.604
	CD19 cell count at diagnosis (cell/mm^3^)	192.68 (144.50)	237.26 (198.43)	0.665
	Last follow-up CD19 cell count (cell/mm^3^)	188.76 (155.24)	185.66 (191.21)	0.282
Immunosuppressant therapy		20 (52.63)	74 (35.97)	0.027*
	Corticosteroids	17 (44.74)	68 (32.23)	0.134
	Azathioprine	7 (18.42)	22 (10.43)	0.168
	Tacrolimus	1 (2.63)	6 (2.84)	1
	Rituximab	8 (21.05)	21 (9.95)	0.056

Mean and SD will be applied for quantitative variables, while N and percentage will be applied for qualitative variables.

* statistically significant.

In the Spanish GTEM-SEMI-CVID Registry, clearly-defined monogenic disorders under a CVID phenotype were excluded. However, analysis of genetic data revealed several variants of uncertain significance (VUS) across a range of genes associated with CVID. The most frequently observed VUS were in the TACI and NFKB1 genes, each identified in 7 patients. The CTLA4 gene was the next most frequently affected, with VUS identified in 5 patients, followed by MBL2, which showed mutations in 3 patients. Variants in IKAROS were noted in 2 patients. Additionally, single occurrences of VUS were observed in several genes, including BTK, NFKB2, LRBA, MLL2, PI3KCD, PI3KR1, PCLG2, PTPN2, RAG1, TFC3, and CD27.

**Figure 1:**
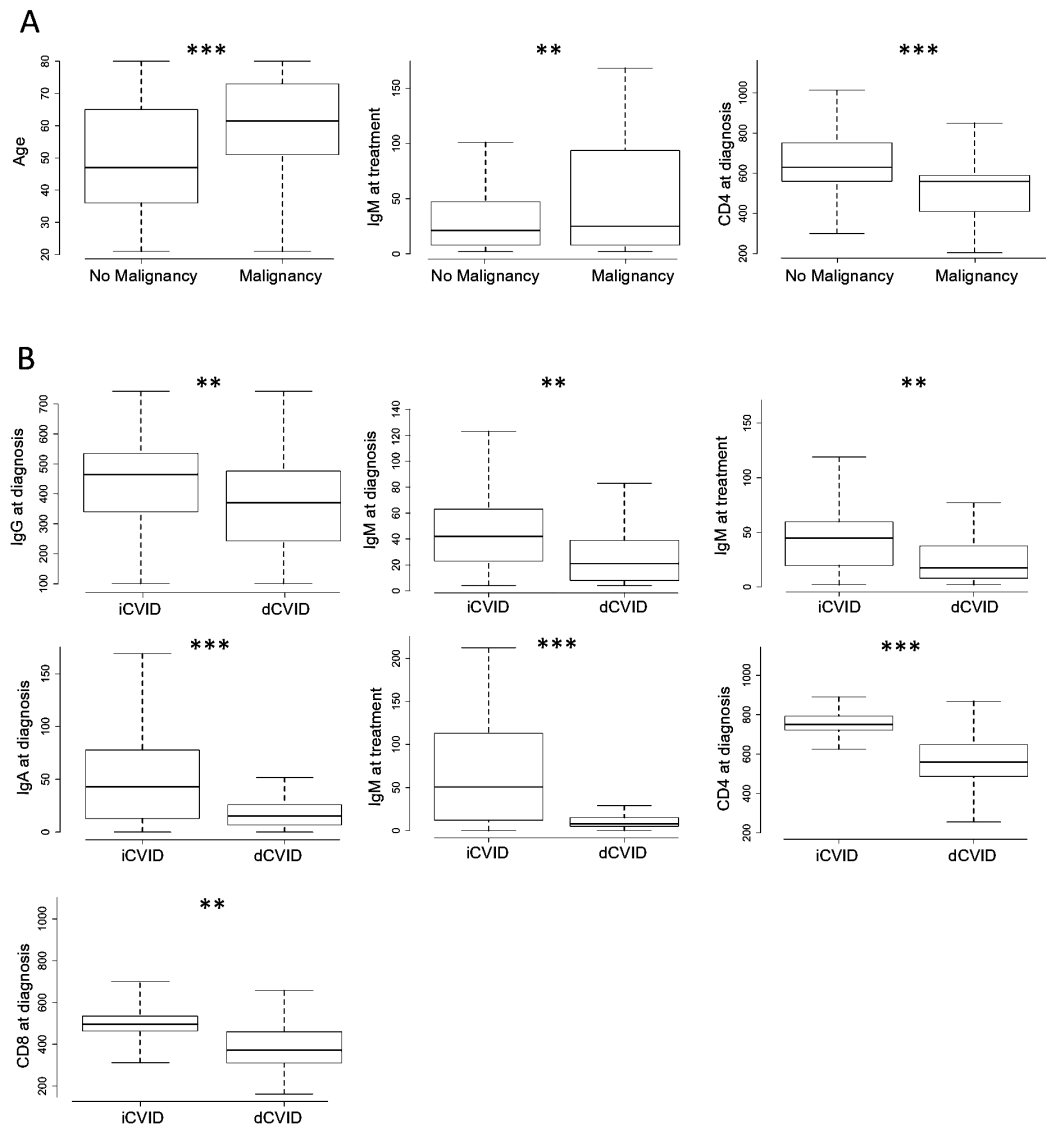
Box plot illustrating the results of the Tukey *post-hoc* test for variables with significant effects, showing **(A)** the impact of immune dysregulation and **(B)** the impact of malignancy. Statistically significant differences are marked as p < 0.05 and **p<0.01, ***p < 0.001.

Cases had received more immunosuppressant therapy than controls during follow-up (55.6% vs. 35.7%, p=0.03). At diagnosis, CD4 cell counts were significantly lower in cases (468.01 cells/µL, SD 243.41) than in controls (715.16 cells/µL, SD 442) (p=0.023), although no differences were observed at the last follow-up. Total IgG, IgA, and IgM levels at diagnosis and during follow-up did not differ between the groups, nor did CD19 or CD8 cell counts. No significant differences were observed in the presence of antinuclear antibodies. However, antineutrophil cytoplasmic antibodies detection was significantly more frequently observed in cancer patients (3.57%) when compared to controls (2.6%) (p=0.023). **Figure 1** displays box plots for those variables where significant main effects of immune dysregulation and malignancy were found (Tukey test).

Patients with immune dysregulation (dCVID) had significantly lower CD4 cell counts at diagnosis (643.57 cells/µL, SD 476.05) compared to patients without immune dysregulation (747.51 cells/µL, SD 306.49) (p=0.016), regardless of the presence of cancer. IgG levels at diagnosis were lower in dCVID patients (383.09 mg/dL, SD 230.97) compared to iCVID patients (440.79 mg/dL, SD 189.53) (p=0.04). IgA levels at diagnosis were also lower in dCVID patients (29.46 mg/dL, SD 44.35) compared to non-dCVID patients (79.30 mg/dL, SD 110.55) (p<0.001). This difference persisted at follow-up (45.72 mg/dL, SD 264 vs. 126.96 mg/dL, SD 235.92, p=0.007). There were no significant differences in IgM levels or CD19 and CD8 cell counts at diagnosis and last follow-up. These results were observed in the two-way ANOVA analysis, but no significant interaction was found for any variable, as seen in **Table 2**.

**Table 2:** Two-way ANOVA analysis assessing the effects of malignancy and immune dysregulation, as well as their interaction, on various clinical and immunological variables in CVID patients.

Variable	Immune dysregulation effect	Malignancy effect	Malignancy x Immune dysregulation interaction
Age	F(1,245) = 2.4	F(1,245) = 14.3***, ηp^2^ = 0.05	F(1,245) = 1.4
IgG levels at diagnosis (mg/dL)	F(1,245) = 10.3** ηp2 = 0.037	F(1,245) = 0.2	F(1,245) = 0.1
Last follow-up IgG levels (mg/dL)	F(1,245) = 0	F(1,245) =0	F(1,245) = 0.9
IgM levels at diagnosis (mg/dL)	F(1,245) = 8.2**, ηp2 = 0.037	F(1,245) =0.11	F(1,245) = 0.07
Last follow-up IgM levels (mg/dL)	F(1,245) = 9.2**, ηp^2^ = 0.04	F(1,245) =8.7**, ηp^2^ = 0.03	F(1,245) = 0.07
IgA levels at diagnosis (mg/dL)	F(1,245) = 30.6***, ηp^2^ = 0.1	F(1,245) = 0.01	F(1,245) = 0.01
Last follow-up IgA levels (mg/dL)	F(1,245) = 61.3***, ηp^2^ = 0.2	F(1,245) = 0.2	F(1,245) = 0.1
CD4 cell count at diagnosis (cell/mm^3^)	F(1,245) = 30.1***, ηp^2^ = 0.09	F(1,245) = 7.4**, ηp^2^ = 0.02	F(1,245) = 0.14
Last follow-up CD4 cell count (cell/mm^3^)	F(1,245) = 0.3	F(1,245) =0.06	F(1,245) = 0.23
CD8 cell count at diagnosis (cell/mm^3^)	F(1,245) = 6.3*, ηp^2^ = 0.02	F(1,245) = 0.9	F(1,245) = 0.001
Last follow-up CD8 cell count (cell/mm^3^)	F(1,245) = 3.1	F(1,245) = 0.4	F(1,245) = 0.003
CD19 cell count at diagnosis (cell/mm^3^)	F(1,245) = 0.07	F(1,245) =0.8	F(1,245) = 0.03
Last follow up CD19 cell count (cell/mm^3^)	F(1,245) = 0.1	F(1,245) =0.001	F(1,245) = 0.1

The table presents the F-values and significance levels from a Two-Way ANOVA and highlighted in bold examining the effects of malignancy, immune dysregulation, and their interaction on various clinical and immunological variables in CVID patients. Significant results are marked with asterisks and highlighted in bold (*p < 0.05, **p < 0.01, ***p < 0.001), indicating a meaningful impact of the factor on the variable. Higher F-values represent a stronger effect. Additionally, partial eta squared (ηp²) is included to indicate the proportion of variance in the data explained by immune dysregulation or malignancy, with higher values representing greater effect sizes. For example, in IgA levels at diagnosis, ηp² = 0.1, suggesting that 10% of the variability in IgA levels is due to immune dysregulation.

### 3.2 Multivariable regression analysis

**Figure 2:**
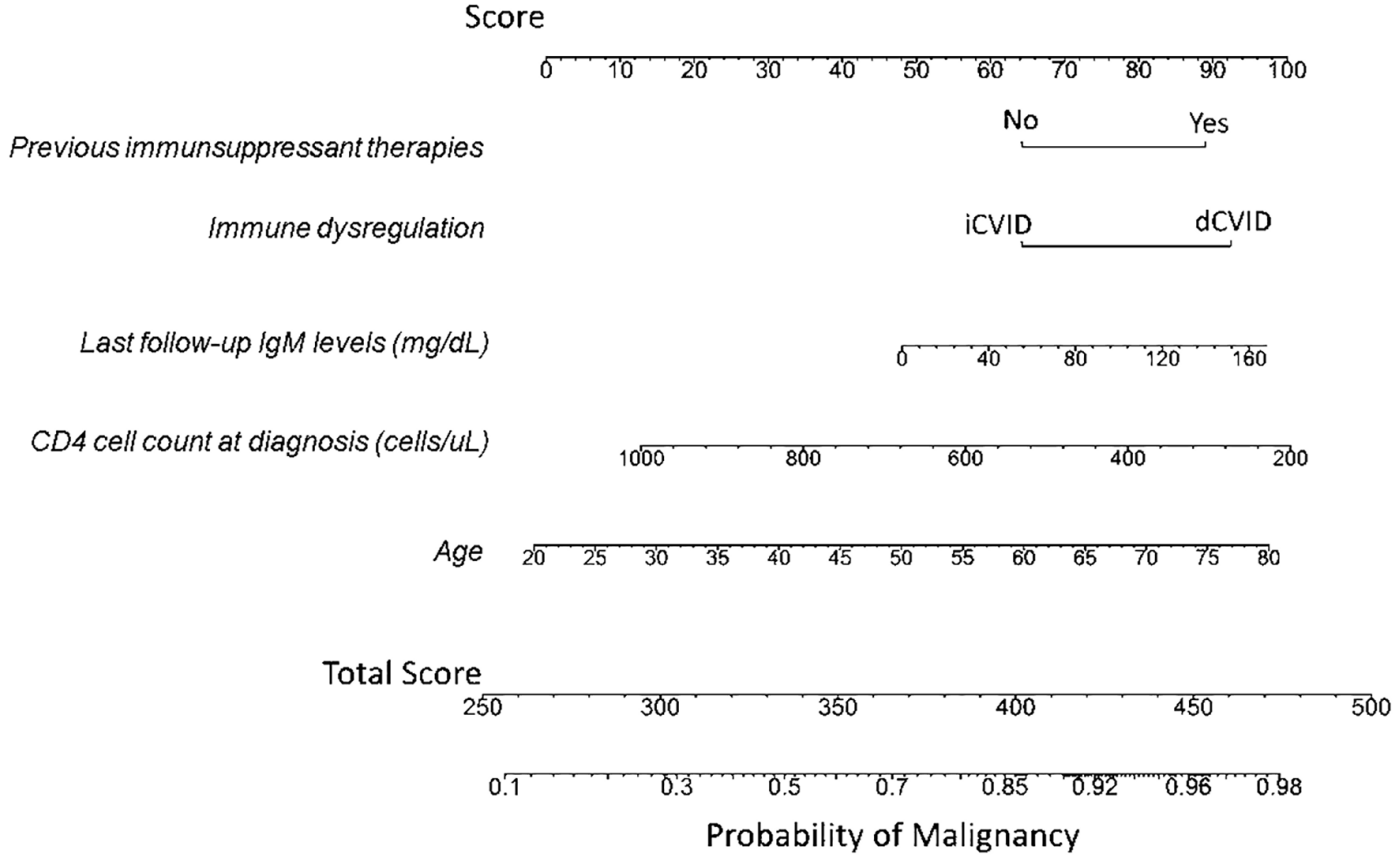
Nomogram illustrating the predicted probability of malignancy based on the predictive risk factors. The total score corresponds to the probability of malignancy occurring, shown on the bottom scale. To obtain the nomogram-predicted probability of malignancy, first locate the patient’s value for each of the five variables on their respective axes. Draw a vertical line from each variable value to the upper “Score” axis to determine the points attributed to each value. Sum the points for all variables, and then locate the total points on the “Total Score” axis. Finally, draw a vertical line from the total points to the “Probability of Malignancy” axis to determine the estimated probability of malignancy. Example 1: A 65-year-old patient with CVID presented with immune dysregulation manifested as immune thrombocytopenic purpura (ITP) requiring immunosuppressive therapy. The patient had an IgM level under IgRT of 125 mg/dL and a CD4 cell count at diagnosis of 350 cells/µL. For dCVID associated with immune dysregulation, plotting a vertical line to the “Score” axis yields approximately 92 points. Similarly, an age of 65 corresponds to 74 points, the use of immunosuppressants to 89 points, an IgM level of 125 mg/dL to 85 points, and a CD4 cell count of 350 cells/µL to about 90 points. The total score for this patient is 432 (92 + 74 + 89 + 85 + 90). Using this total score on the “Total Score” axis and drawing a vertical line to the “Probability of Malignancy” axis indicates an estimated malignancy risk of approximately 92%. Example 2: In contrast, a 25-year-old CVID patient without immune dysregulation, who has never received immunosuppressive therapy, has an IgM level under IgRT of 40 mg/dL and a CD4 cell count at diagnosis of 700 cells/µL. The absence of immune dysregulation corresponds to 62 points on the upper score line, an age of 25 to approximately 8 points, no history of immunosuppressive therapy to 63 points, an IgM level of 40 mg/dL to 60 points, and a CD4 cell count of 700 cells/µL to about 45 points. The total score for this patient is 238 (62 + 8 + 63 + 60 + 45), corresponding to a malignancy probability of less than 10%.

A multivariable logistic regression analysis was conducted to assess the contribution of various parameters to cancer occurrence in our cohort. Initially, all potential predictors for cancer, as outlined in the Methods section, were filtered based on VIF, data sparsity, and biological plausibility. The final model included the variables age, immunosuppressant therapy, immune dysregulation, IgM levels at last follow-up and CD4 cell count at diagnosis, which showed each a significant univariant relationship with malignancy occurrence and therefore were considered as independent associates of malignancy in our cohort. The logistic model was significant with respect of the null-model (Likelihood Ratio test, p<0.001). The ORs, the confidence interval of the ORs and the p-values obtained from the Wald test applied to each coefficient are reported in **Table 3**. Immune dysregulation increased the odds of cancer by 2.19 times (p=0.04), and previous immunosuppressant therapies raised the odds by 2 (p=0.031). Increases in IgM levels in one standard deviation (203.3 mg/dL) raised the odds by 1.008 times (p=0.012). Furthermore, age and CD4 cell count at diagnosis were considered as risk factors with ORs of 1.04 (p<0.001) and 0.997 (p<0.001). Nomogram associated to this model is shown in **Figure 2**.

**Table 3:** Logistic regression analysis of the occurrence of Malignancy. Odds-ratio, p-value and the 95% confidence interval of each independent variable are shown.

Variable	OR	CI 95%	P-value
Intercept	0.13	0.02-0.66	0.014
Immunosuppressant treatment	2	1.06-3.79	0.031
Immune dysregulation	2.19	1.01-4.88	0.04
Age	1.04	1.02-1.06	<0.001
Last follow-up IgM levels	1.008	1.001-1.01	0.012
CD4 cell count at diagnosis	0.997	0.995-0.998	<0.001

**Figure 3:**
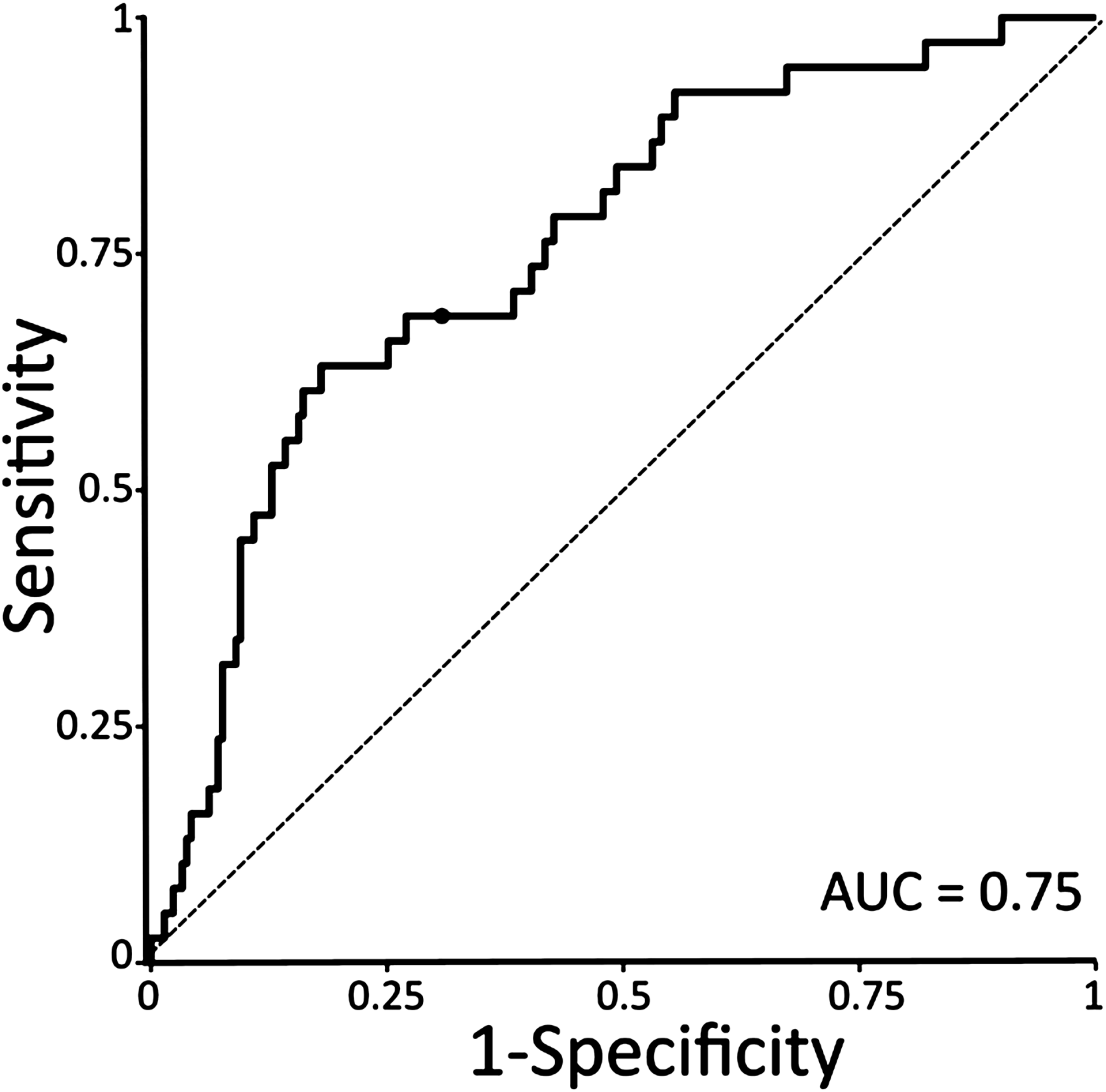
Receiver Operating Characteristic (ROC) curve illustrating the performance of the logistic regression model in distinguishing between Malignancy and No malignancy. The diagonal dashed line represents the baseline performance of a random classifier. Dot point shows the optimal cut-off point based on the maximal specificity and sensibility, which corresponds to 0.5.

We performed a leave-one-out cross-validation to assess the predictive performance of this exploratory model. The model’s predictive performance, evaluated by the AUC, was 0.75 (**Figure 3**). The model showed an overall accuracy of 0.69, with a sensitivity of 0.68 and a specificity of 0.69.

## 4 Discussion

In this study, we investigated the risk factors for cancer development in a cohort of 249 CVID patients, with particular emphasis on immune dysregulation. In the GTEM-SEMI-CVID Registry, older age, immune dysregulation, previous immunosuppressant therapies, higher IgM levels, and lower CD4 cell counts at diagnosis were independently associated to malignancy in CVID from a multivariable approach (**Figure 2**).

Patients with CVID have a significantly increased risk of both hematological and solid malignancies, with a reported prevalence of approximately 10% (ranging from 1.5% to 20.7%). In our registry, the prevalence of cancer was 15.26%, one of the highest reported rates (5, 6, 8, 9, 15, 16), and significantly higher than the estimated global cancer prevalence of 1-2% in the general Spanish population aged 45-54. Consistent with prior studies, non-Hodgkin B-cell lymphoma was the most common malignancy, with a frequency of 4.4%, which is also higher than the global prevalence of non-Hodgkin lymphoma in the Spanish population for that same age range, estimated at 0.01-0.02%. This was followed by gastric cancer, lung tumors, and cutaneous malignancies.

Despite the evidence is very limited to date, some works suggest that immune dysregulation could be associated with the development of cancer both in pediatric (11) and adult CVID patients (12, 13). Several specific manifestations of this dysregulation have been identified as potential risk factors for malignancy. Namely, in a Czech study following 295 patients of which 22 developed cancer, history of Immune thrombocytopenic purpura (ITP) was established as a potential risk factor, with over 3 times higher risk of cancer development (14). Moreover, immune dysregulation manifesting as arthritis, atrophic gastritis, or interstitial lung disease (ILD) was associated with a cancer diagnosis in a German cohort of 219 patients, including 27 with cancer (17). However, the epidemiological profile of this population differs markedly from that of the Mediterranean, where arthritis is very uncommon (7).

Interestingly, our findings concur with some studies suggesting that elevated IgM is associated with an increased risk of malignancies, including lymphoma, in CVID patients. Several years ago, Resnick et al. (5) reported that higher IgM levels were associated with reduced survival in CVID, in the context of increased mortality rates associated with lymphoma. Indeed, in some subgroups of CVID patients, this IgM elevation has been suggested as a condition-specific marker of lymphoma (18). Other studies have reported this association also with lymphoproliferation and high risk of malignancies (19–21). The increase in this biomarker has also been linked to immune dysregulation, such as in interstitial lung disease and pulmonary B-cell hyperplasia (22).

The relationship between cancer and lower CD4 cell counts in CVID is not clearly established, as we have found in our cohort. However, some studies suggest that lower numbers and impaired function of CD4 T cells and natural killer cells are linked to a higher risk of malignancy in these patients (21, 23) by increasing inflammation, accelerating immunosenescence, and impairing immune surveillance (24). Importantly, an early decline in CD4 T cell counts may prolong the timeframe for neoplasia development, which could be particularly relevant for dCVID patients who present with lower CD4 T cell counts at diagnosis. Moreover, some reports of patients with CVID and several solid and hematologic neoplasia have highlighted very low CD4/CD8 ratios (25). These lower counts have also been more frequently found in a subgroup of CVID patients exhibiting increased autoimmunity, granulomas, splenomegaly, and expanded CD21low B cells (26, 27), which, in our cohort, was also independently associated to an increased risk of cancer. However, evidence could be more consistent regarding immune markers (17).

In this context, the association between immune dysregulation and higher IgM levels linked to persistent inflammation, a reduced CD4 T cell compartment, and the use of immunosuppressive therapies to manage these complications, synergistically contributes to the development of neoplasia. Although these treatments are necessary for controlling immune dysregulation, they have been shown to be independently associated to neoplasia in this cohort. Therefore, their use requires caution, coupled with a higher index of suspicion and more comprehensive screening measures.

Despite the association of iatrogenic immunosuppression to malignancy in other patient subgroups such as solid organ transplant recipients is well-studied, evidence is lacking in CVID patients and longitudinal data are strongly needed to guide management (28). Previous efforts have focused on infectious comorbidities in these both primarily and secondarily immunocompromised CVID individuals (7, 29). However, prospective analyses of neoplasia in these patients are especially important given the multifactorial interplay of genetics, immune dysregulation, and chronic infectious agents, including oncogenic microorganisms such as Epstein–Barr virus (EBV) (30) and *H. pylori* (7), which may be even more prevalent in this doubly immunocompromised population.

This nationwide, multicenter study systematically compiles extensive data on CVID patients, using advanced statistical methods to identify independent malignancy associates in a large CVID cohort under a robust temporal framework for data collection. However, it is not exempt from limitations. The retrospective cross-sectional nature of this work is an inherent bias as it limits the ability to establish causal relationships between predictors and malignancy development, and we can talk about potential associates that should be further explored in future prospective studies that can more rigorously test these hypotheses, both from clinical or basic and translational approaches. Furthermore, the study’s focus on Spain might introduce unique environmental and genetic factors affecting cancer prevalence and associated factors. The exclusion of patients with confirmed monogenic immunodeficiencies may reduce the generalizability of the findings to all CVID patients, especially considering some monogenic mutations could be linked to higher susceptibility to neoplasia, such as in the NFK-B signaling pathway implicated in many hallmarks for carcinogenesis (31). Additionally, the reliance on data from participating hospitals could introduce selection bias, as patients in specialized centers might have more severe disease manifestations. Moreover, the potential for missing data, despite the use of median-based or imputation, may affect the robustness of the statistical analyses. Finally, the retrospective nature of this study allows for the detection of associations but not causal relationships, necessitating prospective studies to establish causality.

## 5 Conclusion

This study identifies key potential predictors of malignancy in CVID patients, including older age, immune dysregulation, previous immunosuppressant therapies, elevated IgM levels, and lower CD4 cell counts at diagnosis. The findings underscore the heightened cancer risk in this population and highlight the necessity for vigilant monitoring and tailored screening protocols to improve patient outcomes, especially in those with increased inflammation and immune dysregulation. Further prospective research is needed to establish causality, enhance management strategies for CVID-associated malignancies, and deepen the molecular and genomic understanding of the underlying pathophysiology and common carcinogenic signaling pathways also implicated in CVID.

## Data Availability

The raw data supporting the conclusions of this article will be made available by the authors, without undue reservation.
